# Reduction of graphene oxide by an *in-situ *photoelectrochemical method in a dye-sensitized solar cell assembly

**DOI:** 10.1186/1556-276X-7-101

**Published:** 2012-02-02

**Authors:** Chen Chen, Mingce Long, Min Xia, Chunhua Zhang, Weimin Cai

**Affiliations:** 1 School of Environmental Science and Engineering, Shanghai Jiao Tong University, Dongchuan Road 800, Shanghai 200240, People's Republic of China

**Keywords:** graphene oxide, reduction, photoelectrochemical, *J*-*V *curve, dye-sensitized solar cells

## Abstract

Reduction of graphene oxide [GO] has been achieved by an *in-situ *photoelectrochemical method in a dye-sensitized solar cell [DSSC] assembly, in which the semiconductor behavior of the reduced graphene oxide [RGO] is controllable. GO and RGO were characterized by X-ray photoelectron spectroscopy, Raman spectroscopy, high-resolution transmission electron microscope, and Fourier-transform infrared spectroscopy. It was found that the GO film, which assembled in the DSSC assembly as the counter electrode, was partly reduced. An optimized photoelectrochemical assembly is promising for modulating the reduction degree of RGO and controlling the band structure of the resulting RGO. Moreover, this method appeared to be a green progress for the production of RGO electrodes.

## Introduction

Nowadays chemical conversion of solar energy has attracted considerable attention [1-3]. Graphene is a new carbon material with diverse properties being suitable for energy conversion and storage [4]. Graphene oxide [GO], produced by exfoliation of graphite oxide, has been traditionally considered to be a precursor for graphene [5-7]. GO has recently attracted research interest due to its good solubility in water and other solvents, which allows it to be easily deposited onto a wide range of substrates [8,9]. Besides, GO has variable optical, mechanical, and electronic properties that can be tuned by controlling the degree of oxidation [10,11]. Reduced graphene oxide [RGO], characterized as an incompletely reduced product of GO, is the intermediate state between graphene and GO. Because the oxygen bonding forms the *sp*^3 ^hybridization on RGO [12] and oxygen atoms have a larger electronegativity than carbon atoms, RGO becomes a doped semiconductor where the charge flow creates negative oxygen atoms and a positively charged carbon grid [13,14]. However, understanding the controllable semiconductor behavior of RGO is still a big challenge. Normally, the bandgap of RGO increases with the oxidation level. Controlling the ratio of *sp*^2 ^carbon atoms to *sp*^3 ^carbon atoms by reduction chemistry is a powerful way to tune its bandgap. Therefore, RGO can be controllably transformed from an insulator (GO) to a conductor (graphene) [10,15]. Owing to these characteristics, RGO has great potential to be applied in biosensors [16], optical devices [17], plastic electronics [18], and solar cells [19]. The reduction of GO is typically achieved by thermal annealing and exposure to hydrazine gas, as described in former cases [20-22]. These methods involved either high temperatures or a poisonous and explosive gas.

In this work, we present a green and controllable approach for the *in-situ *reduction of GO in a dye-sensitized solar cell [DSSC] assembly. The GO film was fabricated in the DSSC as a counter electrode. In typical DSSCs, upon illumination, photoinduced electrons from the excited dye transfer toward the conduction band of TiO_2 _photoanodes, accompanying the oxidation of redox species in the electrolyte (e.g., I^-^/I_3_^-^), and simultaneously, the reduction reaction occurs at the counter electrodes by accepting the electrons. By substituting the Pt counter electrode with the GO film, the photoinduced electrons could be captured by GO and result in the reduction of GO. Inspired by this, in this contribution, we provide an easy approach to an *in-situ *photoelectrochemical reduction of GO with a GO drop-cased fluorine-doped tin oxide [FTO] glass as the counter electrode in a DSSC assembly. Moreover, according to the transition mechanism, an optimized photoelectrochemical assembly can be fabricated for the controllable modulation of the band positions of RGO materials.

### Experimental section

#### Preparation of GO

GO was synthesized from natural graphite powder (100 μm; Qingdao Graphite Company, Qingdao, Shandong, China) by a modified Hummers' method [23]. In a typical experiment, the graphite powder (1 g) and NaNO_3 _(0.5 g) were introduced to concentrated H_2_SO_4 _(23 mL) in an ice bath. KMnO_4 _(3 g) was added gradually under stirring to prevent rapid temperature rise, and the temperature of the mixture was kept below 20°C. The mixture was then stirred at 35°C for 4 h. Then, deionized water (46 mL) was slowly added to the solution, followed by stirring the mixture at 98°C for 15 min. The reaction was terminated by adding deionized water (140 mL) and H_2_O_2 _(1 mL, 30 wt.%) under stirring at room temperature. The resulting graphite oxide was washed with deionized water by filtration. Graphene oxide was obtained from the graphite oxide solution by ultrasonication at room temperature for 30 min. Unexfoliated graphite oxide in suspension after ultrasonication was removed by centrifugation at 3,000 rpm for 5 min.

#### Fabrication of DSSCs

To realize the *in-situ *photoelectrochemical reduction, GO counter electrode was used for DSSCs. The GO electrode was prepared by drop casting the GO solution of 1 mg/ml on a clean FTO glass substrate and dried in room temperature. N719-sensitized TiO_2 _film anode was prepared according to the literature method [24]. In brief, 1.6 g of nanocrystalline TiO_2 _and 0.7 g of ethyl cellulose were suspended with 6 mL of terpilenol. Five layers of 20-nm-sized TiO_2 _particles and two layers of 400-nm-sized TiO_2 _particles were screen printed on a TiCl_4_-treated FTO glass. These films were heated to 500°C in air and sensitized with a 0.36 mg/ml N719 dye solution for 24 h. The cell had an active area of 0.36 cm^2 ^and was sealed with an electrolyte solution containing 0.1 M lithium iodide, 0.05 M iodine, 0.5 M 4-*tert*-butylpyridine, and 0.6 M ionic liquid (1, 2-Dimethyl-3-propylimidazolium bis(trifluoromethylsulfonyl)imide).

#### Characterization

Solar conversion efficiency and current density-voltage [*J*-*V*] curves were measured under air mass [AM] 1.5 G light with a solar simulator (HMT Co., Bangalore, India), and a potentiostat (Keithley 2400, Keithley Instruments Inc., Cleveland, OH, USA) was used to apply various loads. The incident light intensity was calibrated using a standard solar cell composed of a crystalline silicon solar cell and an infrared cutoff filter (KG-5, Schott AG, Mainz, Germany).

Raman spectra were obtained on a Senterra R200-L dispersive Raman microscope (Bruker Optik Gmbh, Ettlingen, Germany) with a 633-nm laser source. The morphology and structure were observed by a JEM-2100F high-resolution transmission electron microscope [HRTEM] (JEOL Ltd., Akishima, Tokyo, Japan) operated at 200 kV. Fourier transform infrared [FT-IR] spectroscopy was conducted using a Fourier transform infrared spectrometer (EQUINOX 55, Bruker Optik Gmbh, Ettlingen, Germany). X-ray photoelectron spectroscopy [XPS] experiments were carried out on a RBD-upgraded PHI-5000C ESCA system (PerkinElmer, Waltham, MA, USA) with AlKα radiation (hv = 1486.6 eV).

## Results and discussion

**Figure 1:**
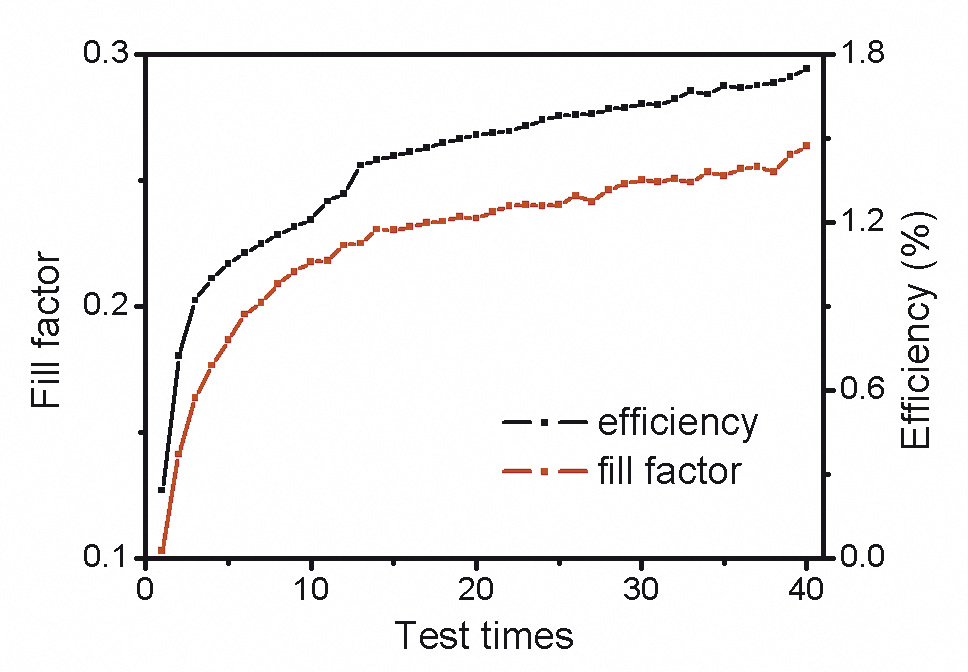
**Fill factor and solar conversion efficiency in different tests**.

**Figure 2:**
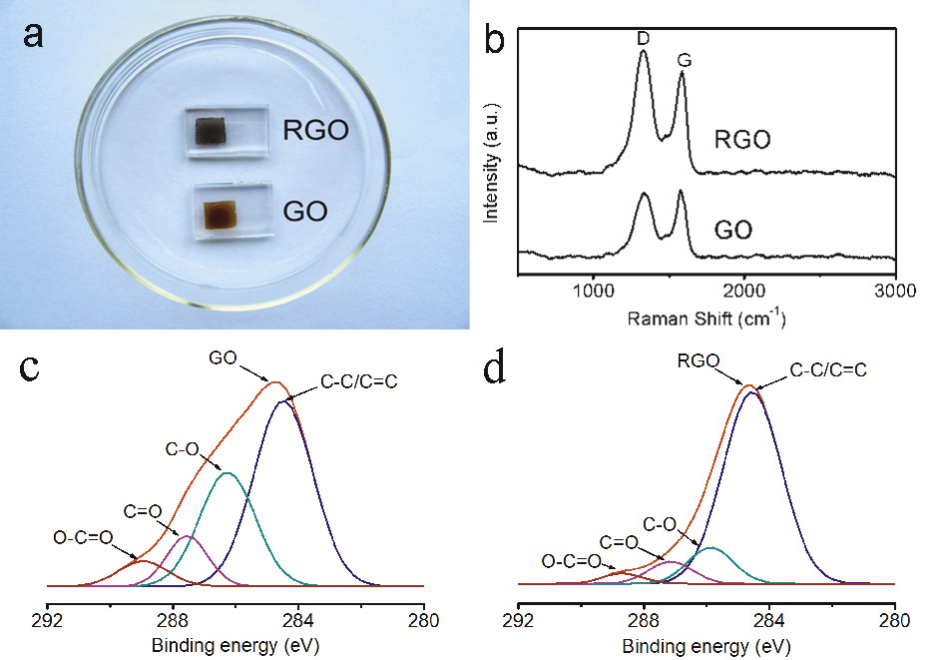
**Digital picture and Raman and XPS spectra**. (**a**) Digital picture of GO and RGO, (**b**) Raman spectra of GO and RGO, and (**c**) C1*s *XPS spectra of GO before and (**d**) after reduction.

The *J*-*V *characteristics of DSSCs were taken under AM 1.5 G light. The changes of fill factors [FF] and solar conversion efficiencies [*η*] in 40 tests were tracked and shown in Figure 1. Upon illumination during the *J*-*V *tests, FF and *η *increase sharply on the first few tests. It is due to the reduction of the GO film by the photoinduced electrons. With the reduction proceeding, the increase of FF and *η *slows down and approaches to a limitation at last. After the photoelectrochemical reduction, the GO film on the counter electrode is reduced into RGO. The FF and *η *enhance from 0.10 and 0.24% to 0.28 and 1.75%, respectively, indicating that a significantly improved conductivity of the RGO film has been achieved. After the reduction, the RGO counter electrode was immersed in acetonitrile for 1 h to remove the adsorbed I^- ^and I_3_^- ^and dried with N_2 _flow for further characterization. From the images of Figure 2a, the color of the film changed obviously from brown to black, indicating that a reduction was achieved for the GO electrode.

To confirm the reduction of GO, Raman spectroscopy, a powerful nondestructive tool to characterize crystal structures of carbon, was employed. The typical features for carbon in Raman spectra are the G line around 1,582 cm^-1 ^(*E*_2*g *_phonon of *sp*^2 ^carbon atoms) and D line around 1,350 cm^-1 ^(*κ*-point phonons of *A*_1*g *_symmetry). Figure 2b shows the Raman spectra of GO and RGO. The intensity ratio (*I*_D_/*I*_G_) is about 0.96 for GO, while the ratio of RGO is much higher (1.27). Comparing with the results by chemical reduction methods, such as NaBH_4 _(> 1) [22], hydrazine hydrate (1.63) [25], and hydrothermal reduction (0.90) [26], the high ratio of 1.27 implies that GO on the FTO glass was reduced significantly by the photoinduced electrons in the DSSC.

The reduction of GO was described by X-ray photoelectron spectroscopy as well. The C1*s *spectrum of the original GO film (Figure 2c) reveals that there are four different peaks centering at 284.4, 286.0, 287.1, and 288.7 eV, corresponding to C=C/C-C, C-O (hydroxyl and epoxy), C=O (carbonyl), and O-C=O (carboxyl) groups, respectively. After the *in-situ *photoelectrochemical reduction in the DSSC, the peaks of oxygen-containing groups, especially the peak of C-O, decrease dramatically (shown in Figure 2d), and the percentages of oxygen-containing groups are shown in Table 1. The results reveal that most of the oxygen-containing groups are removed. In addition, the atomic ratio of carbon and oxygen (C/O), obtained by taking the ratio of C1*s *and O1*s *peak areas in XPS spectra, increases from 2.7 to 5.1, which also indicates the reduction of GO did take place during the *in-situ *reduction in the DSSC.

**Table 1:** Percentages of oxygen-containing groups of GO and RGO from XPS data

Sample	C-O	C=O	O-C=O
GO	20.8	10.0	6.1
RGO	11.5	6.9	2.9

**Figure 3:**
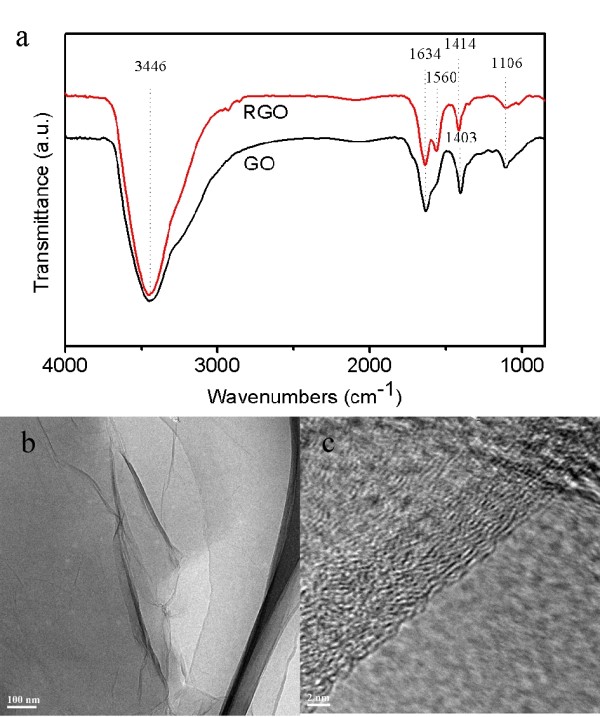
**FT-IR spectra and TEM and HRTEM images**. (**a**) FT-IR spectra of GO and RGO and (**b**) TEM and (**c**) HRTEM images of RGO.

The reduced GO film was removed from the FTO glass for the tests of FT-IR spectroscopy and HRTEM. FT-IR spectroscopy was also used to indicate the reduction of oxygen-containing groups of GO. Figure 3a shows the characteristic bands of GO around 1,106 cm^-1 ^for C-O (ν(alkoxy and epoxy)), 1,403 cm^-1 ^for O-H (ν(carboxyl)), 1,634 cm^-1 ^for C=C, and 3,446 cm^-1 ^for O-H of intercalated water. After the reduction, the absorption bands of both C-O and O-H are considerably decreased. The characteristic band of C=O (carboxyl) was not detected by FT-IR. The results imply that most of the oxygen-containing groups of GO were reduced by the photoinduced electrons.

RGO obtained by *in-situ *reduction was analyzed by HRTEM. Figure 3b shows a low magnification image of a typical RGO nanosheet. The sheets resemble crumpled silk veil waves on the carbon-coated copper grid. As reported previously, corrugation and scrolling are intrinsic to graphene nanosheets [27]. The ordered graphene lattices are clearly visible in the HRTEM image of RGO (Figure 3c). A 0.39-nm intersheet spacing obtained from this image indicates a moderate oxidation level of RGO because the layer distance of typical oxidized graphite is between 0.6 to 0.7 nm [28].

**Figure 4:**
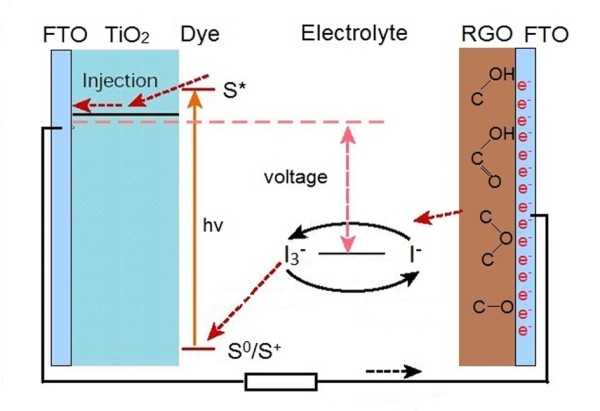
**Mechanism of *in-situ *photoelectrochemical reduction of GO in a DSSC assembly**.

According to the results of characterization on the GO film before and after reduction, it was confirmed that the GO film was partly reduced when the DSSC was exposed upon irradiation. A scheme for the *in-situ *photoelectrochemical reduction of the GO film was depicted in Figure 4. As we know, GO/RGO can be regarded as semiconductors with changeable energy band positions depending on the ratios of *sp*^2 ^carbon to *sp*^3 ^carbon [9,10]. Initially, the reduction potential of photoinduced electrons at the counter electrode is powerful enough to reduce GO and makes its oxidation level reduced. Moreover, electrons are not easy to be captured efficiently by the ions I_3_^- ^due to the relatively higher oxidation power and lower conductivity of GO, resulting in lower conversion efficiencies and higher open voltages at the first few measurements. With the reduction of GO, the atomic ratio of carbon to oxygen increases and the bandgap of GO decreases; meanwhile, the valence band of GO shifts upward. With the increased conductivity of GO, *J*_SC_, FF, and *η *of the as-assembled DSSC rise continuously. However, the reduction decelerates and ceases when the valence band of RGO shifts to the position which is higher than the reduction potential of I^-^/I_3_^-^. By then, electrons are captured by I_3_^-^; meanwhile, the efficiency of the DSSC no longer increases. That is the reason for the incomplete reduction of GO and the relatively lower solar conversion efficiency of the resulting DSSC. However, this method is convenient to obtain a RGO film with different reduction degrees. Moreover, it provides a promising strategy for modulating the band positions of RGO.

## Conclusions

RGO is obtained by the *in-situ *photoelectrochemical reduction of GO in a DSSC assembly. The reduction results in a partial removal of oxygen-containing groups of GO. This method avoids the high-temperature processing and the usage of harmful chemical reagents. The reduction of GO is changeable by controlling the irradiation time or substituting the reduction couples of I^-^/I_3_^- ^in the electrolyte; a further improved performance can be achieved by the optimized photoelectrochemical assembly.

## Competing interests

The authors declare that they have no competing interests.

## Authors' contributions

CC carried out the total experiment and wrote the manuscript. MCL supervised all the study and performed the statistical analysis. MX participated in the detection of the TEM and FT-IR. CHZ participated in the detection of the *J*-*V *curves and DSSC assemblage. WMC participated in the design of the study and mechanism analysis. All authors read and approved the final manuscript.
